# Individual aptitude in Mandarin lexical tone perception predicts effectiveness of high-variability training

**DOI:** 10.3389/fpsyg.2014.01318

**Published:** 2014-11-25

**Authors:** Makiko Sadakata, James M. McQueen

**Affiliations:** ^1^Centre for Cognition, Donders Institute for Brain, Cognition and Behaviour, Radboud University NijmegenNijmegen, Netherlands; ^2^Institute for Logic, Language and Computation, University of AmsterdamAmsterdam, Netherlands; ^3^Behavioural Science Institute, Radboud University NijmegenNijmegen, Netherlands; ^4^Max Planck Institute for PsycholinguisticsNijmegen, Netherlands

**Keywords:** perceptual learning, variability, lexical tones, L2 learning, individual differences

## Abstract

Although the high-variability training method can enhance learning of non-native speech categories, this can depend on individuals’ aptitude. The current study asked how general the effects of perceptual aptitude are by testing whether they occur with training materials spoken by native speakers and whether they depend on the nature of the to-be-learned material. Forty-five native Dutch listeners took part in a 5-day training procedure in which they identified bisyllabic Mandarin pseudowords (e.g., asa) pronounced with different lexical tone combinations. The training materials were presented to different groups of listeners at three levels of variability: low (many repetitions of a limited set of words recorded by a single speaker), medium (fewer repetitions of a more variable set of words recorded by three speakers), and high (similar to medium but with five speakers). Overall, variability did not influence learning performance, but this was due to an interaction with individuals’ perceptual aptitude: increasing variability hindered improvements in performance for low-aptitude perceivers while it helped improvements in performance for high-aptitude perceivers. These results show that the previously observed interaction between individuals’ aptitude and effects of degree of variability extends to natural tokens of Mandarin speech. This interaction was not found, however, in a closely matched study in which native Dutch listeners were trained on the Japanese geminate/singleton consonant contrast. This may indicate that the effectiveness of high-variability training depends not only on individuals’ aptitude in speech perception but also on the nature of the categories being acquired.

## INTRODUCTION

Speech in everyday life is noisy: there is high variability due, for example, to differences within- and across-speakers, and to differences across linguistic and environmental contexts. Native listeners can cope with such variability better than non-native listeners (Bradlow and Pisoni, 1999). Having phonologically abstract speech categories could help native listeners by facilitating their ability to accommodate to the diversity in the speech signal (Scharenborg et al., 2005; McQueen et al., 2006). Formation of new abstract speech categories would therefore be beneficial when a listener is trying to master a foreign language (L2). Interestingly, variability appears to help listeners to learn new, non-native categories. It has been shown for a variety of non-native contrasts (e.g., English /r/-/l/ by native speakers of Japanese, Logan et al., 1991; Mandarin lexical tones by native speakers of English, Wang et al., 1999; Japanese geminate consonants by native speakers of Dutch, Sadakata and McQueen, 2013). Exposure to a wider range of exemplars appears to enhance the process of building robust and abstract categories and hence to support generalization of learning (Logan et al., 1991; Sadakata and McQueen, 2013).

High-variability training, however, is not always helpful in non-native phonological learning. Recent studies have identified several factors that modulate the benefit of high-variability training, such as the listener’s aptitude (Perrachione et al., 2011), the type of materials (Kingston, 2003; Wade et al., 2004), and the age of participants (Giannakopoulou et al., 2013). The aim of the current study was to examine whether stimulus and participant characteristics interact in determining what kind of training is most beneficial in speech learning. In particular, we asked if the nature of the speech categories being learned determines the effectiveness of high-variability training and whether that, in turn, depends on listener aptitude in speech perception.

Our focus was on perceptual learning of Mandarin Chinese lexical tones by native speakers of Dutch. Mandarin uses four types of pitch contours to distinguish word meanings. A famous example is the syllable ‘ma’ with four tones with different pitch contours representing mother (T1, level tone), hemp (T2, rising tone), horse (T3, rising tone with a dipping contour in the middle), and scold (T4, falling tone). Learning to perceive and distinguish these tonal categories is challenging for native speakers of non-tonal languages (Wang et al., 1999; Halle et al., 2004). In recent years, individual differences in perceptual learning of non-native lexical tones have received considerable attention. These studies have identified several predictors of success in speech learning, such as perceptual aptitude (Perrachione et al., 2011) and working memory ability (Linck et al., 2013), and have revealed that differences in learning performance are associated with differences in the neural correlates of pitch information processing (Wong et al., 2007a, 2008; Chandrasekaran et al., 2012; Sheppard et al., 2012). Notably, Perrachione et al. (2011) demonstrated a cross-over interaction between individuals’ aptitude in speech perception and the benefit of variability in the training material: high-variability training enhanced perceptual learning in high-aptitude perceivers but it harmed learning in low-aptitude perceivers.

There are two reasons to question the generalizability of this finding. First, Perrachione et al. (2011) used artificial monosyllabic stimuli (pseudowords recorded by native speakers of English, on which synthetic pitch contours were superimposed). It is thus not clear whether the results would replicate with natural rather than artificial stimuli. In the present study, therefore, the materials were recorded by native Mandarin speakers. Furthermore, we used bisyllabic instead of monosyllabic stimuli: in the real-world setting of learning and using Mandarin, listeners need to be able to distinguish the tonal categories of individual syllables in the context of the tonal information about neighboring syllables. While bisyllabic nonsense sequences are not the equivalent of everyday Mandarin sentences, they do at least include a tonal context that is lacking in isolated monosyllables.

Second, other studies have shown that the benefits of high-variability training depend on the nature of the to-be-learned categories. Kingston (2003) and Wade et al. (2004) observed that the benefit of high-variability depends on the type of vowel contrast being learned. In particular, Wade et al. (2004) report that the benefit diminishes for more confusing vowel contrasts relative to less confusing ones. This raises the possibility that the effect of listener aptitude on the effectiveness of high-variability training observed by Perrachione et al. (2011) could also depend on the contrast being learnt.

It could be that aptitude matters for non-native categories which are hard to learn, but not for those which are easier to acquire. Indeed, there is some evidence that this is the case. Sadakata and McQueen (2013) observed an overall benefit of high-variability training for the learning of the Japanese geminate/singleton consonant contrast by native speakers of Dutch. They also observed no significant effect of listener aptitude on learning success: there was an overall trend for listeners with greater perceptual aptitude (i.e., those who performed better on a pre-test discrimination task) to improve more over the course of the training. New simple linear regression analyses for each variability group separately (not reported in the original study) confirm the impression given in Figure 6 of the original study that a weak positive relationship between aptitude and improvement was found not only in the high-variability group [as in Perrachione et al., 2011, *t*(14) = 1.613, *b* = 0.687, n.s.] but also in the low-variability group [unlike in Perrachione et al., 2011, *t*(14) = 0.722, *b* = 0.330, n.s.]. These findings indicate that the variability effect in the geminate study was not strongly modulated by listener aptitude, and in particular that there was no cross-over interaction.

It is reasonable to suppose that speakers of non-tonal languages will find it harder to learn tonal categories than to form geminate consonant categories. Several theories have been proposed to account for the relative difficulty of forming non-native speech categories, such as perceptual assimilation theory (Best et al., 2001), the native language magnet model (Kuhl, 1991), and the speech learning model (Frieda et al., 2000). In spite of their differences, these accounts essentially agree that difficulties depend on the relationship between the new L2 speech categories and pre-existing L1 speech categories. There are various types of relationships between L1 and L2 categories. For example, learning can make use of completely new acoustic features to form fully novel categories (e.g., learning of Zulu clicks for native speakers of English; Best et al., 1988). This type is found to be relatively easy to learn, while it is more challenging to learn to adjust the use of acoustic features that are also involved in related and existing L1 category distinctions (e.g., learning that different VOT values distinguish English /b/ and /p/ from Dutch /b/ and /p/; Liberman et al., 1961; Brandmeyer et al., 2012). It is also more challenging when the L2 category contrast is new but the acoustic features are familiar to the listener. In this case, it may matter what the function is that those features play in the native language. For example, segments in Dutch and English do not differ in duration alone. It may thus be relatively easy for native speakers of these languages to learn to use timing information to distinguish Japanese geminates (Amano and Hirata, 2010; Hardison and Motohashi-Saigo, 2010; Tajima et al., 2010; Sadakata and McQueen, 2013). In contrast, although Dutch and English are non-tonal languages, pitch information is used extensively in those languages for intonational purposes (e.g., to signal sentential accents and prosodic grouping, and to indicate whether an utterance is a question). Furthermore, pitch direction is more important in tonal languages, whereas pitch height is more important in non-tonal languages (Guion and Pederson, 2007; Kaan et al., 2007). It may thus be particularly difficult for non-native listeners to learn to use pitch information in a totally different manner. Under these conditions, therefore, listener aptitude may be especially important in determining how effective high-variability training will be. Listeners with high-aptitude in speech perception may be able to reap the benefits of high-variability with hard-to-learn contrasts (i.e., they can use tokens from different talkers to abstract new tonal categories), while those with low-aptitude may be challenged by that same degree of variability.

We therefore carried out a perceptual training experiment in Mandarin, using the same design as that used in the prior Japanese study by Sadakata and McQueen (2013). We trained native Dutch listeners on bisyllabic Mandarin pseudowords with a lexical tone contrast instead of on bisyllabic Japanese pseudowords with a geminate/singleton consonant contrast. In the original study, the effect of two levels of training variability was compared in five sessions. The low-variability group was exposed to more repetitions of a limited set of words spoken by one speaker, while the high-variability group was exposed to fewer repetitions of an extended set of words spoken by multiple speakers. The high-variability materials thus included “acoustic variability” (within- and across-speaker variability) as well as “phonetic variability” (the number of stimuli representing the to-be-learned contrast). The study included a number of subtests: categorization, discrimination of speech materials, and discrimination of non-speech materials. The current study incorporated all these sub-tests. In addition to the difference in type of training materials, we added an extra group who were trained with medium-variability materials in order to closely examine potential interactions between variability and listener aptitude. In order to estimate individuals’ aptitude in perceiving tonal contrasts, we used a measure of perceptual sensitivity derived from the categorization test. Participants were assigned to either a high- or a low-aptitude group based on this measure.

The design allowed us to investigate two questions. First, we could ask whether the interaction between individuals’ aptitude and the benefits/costs of high-variability training as reported in Perrachione et al. (2011) extends to learning of bisyllabic pseudowords spoken by native speakers of Mandarin. We predicted that we would be able to replicate the interaction, and hence that we would observe no overall benefit of high-variability training and instead find the cross-over pattern of benefits of high-variability training for high-aptitude perceivers but costs for low-aptitude perceivers. Note also that Perrachione et al. (2011) taught their participants new words (associations of sequences with tones to meanings) while we trained participants to associate tonal patterns with arbitrary numerical category labels. This study is thus also a test of whether the aptitude-variability interaction is true for different aspects of L2 learning. Second, the similarity of the design to the Japanese consonant-learning study (Sadakata and McQueen, 2013) allowed us to compare the two sets of results directly. As just noted, we predicted no overall benefit of high-variability training, and hence that we would not replicate the findings of the Japanese study. Such a result would support the suggestion that the effectiveness of high-variability training depends on the nature of the categories being acquired, such that listener perceptual aptitude modulates effectiveness more for hard-to-learn contrasts than for easy-to-learn contrasts.

## MATERIALS AND METHODS

The experiment was designed to be as similar as possible to that in Sadakata and McQueen (2013). Five sessions took place with a maximum duration of 2 days between sessions, resulting in a total experiment duration of from 5 to 9 days. Each session included different perceptual subtests and a training phase (presented in the order given in **Table 1**). The training phase employed a 2-alternative forced choice (2AFC) identification task with feedback in which Dutch participants were trained to identify the tone of the first syllable of naturally spoken bisyllabic Mandarin pseudowords (see Training). In particular, participants learned to discriminate T2 (rising tone) from T3 (rising tone with a dipping contour in the middle) in T21 and T31 bisyllables. Pilot testing had established that this was a particularly difficult tonal contrast for Dutch listeners to acquire. In order to assess perceptual learning, identification of natural minimal-trio bisyllabic stimuli took place everyday before and after the training phase (see Pre- and Post- Training Identification Tests). On day 5, a transfer-of-learning identification test was administered to test the generalizability of learning (see Transfer Test). All identification tests employed natural spoken materials. In addition to these tests, we administered two additional tests (2AFC categorization on days 1 and 5, and 4 interval 2 Alternative Forced Choice (4I2AFC) discrimination on days 1, 3, and 5) using synthetic stimuli (see Categorization of Synthesized Stimuli and Discrimination of Synthesized Stimuli). These tests were included in order to measure individuals’ sensitivity to pitch contour information: how sensitive they were prior to training sessions, and if the sensitivity changed after five training sessions. There were three groups of listeners. The only difference among groups was in the materials (and hence the variability in terms of speakers, words, and tokens) presented during the five training phases.

**Table 1:** The five experimental sessions.

Day 1	Day 2	Day 3	Day 4	Day 5
Identification	Identification	Identification	Identification	Identification
Discrimination (s)	Training	Training	Training	Training
Discrimination (n)	Identification	Discrimination (s)	Identification	Transfer
Categorization (s)		Discrimination (n)		Discrimination (s)
Categorization (n)		Identification		Discrimination (n)
Training				Categorization (s)
Identification				Categorization (n)
				Identification
**80 min**	**30 min**	**45 min**	**30 min**	**90 min**

*s, speech materials; n, non-speech materials*.

### PARTICIPANTS

Forty-five native speakers of Dutch were recruited from the participant pool of the Max Planck Institute for Psycholinguistics (29 females and 16 males, average age 21.0 years old). They were randomly assigned to three groups: low-, medium-, and high-variability. Although the majority of the participants spoke multiple languages at different fluency levels, none of them had had substantial exposure to Mandarin. All participants indicated their self-evaluated fluency level on a scale from 1 (not fluent at all) to 5 (very fluent) with regard to each of their L2s. The reported L2s included English (*N* = 45, fluency level 3.5–5), German (*N* = 36, fluency level 1–3), French (*N* = 36, fluency level 1–3), Spanish (*N* = 5, fluency level 1–4), and Hungarian (*N* = 1, fluency level 1). Participants received 60 euros after taking part in five training sessions. All participants provided written consent prior to participation. The research (with non-invasive procedures and consenting adults) was exempt under Dutch legislation for ethical review and was carried out in accordance with the Declaration of Helsinki.

### MATERIALS AND PROCEDURE

#### Training

**FIGURE 1:**
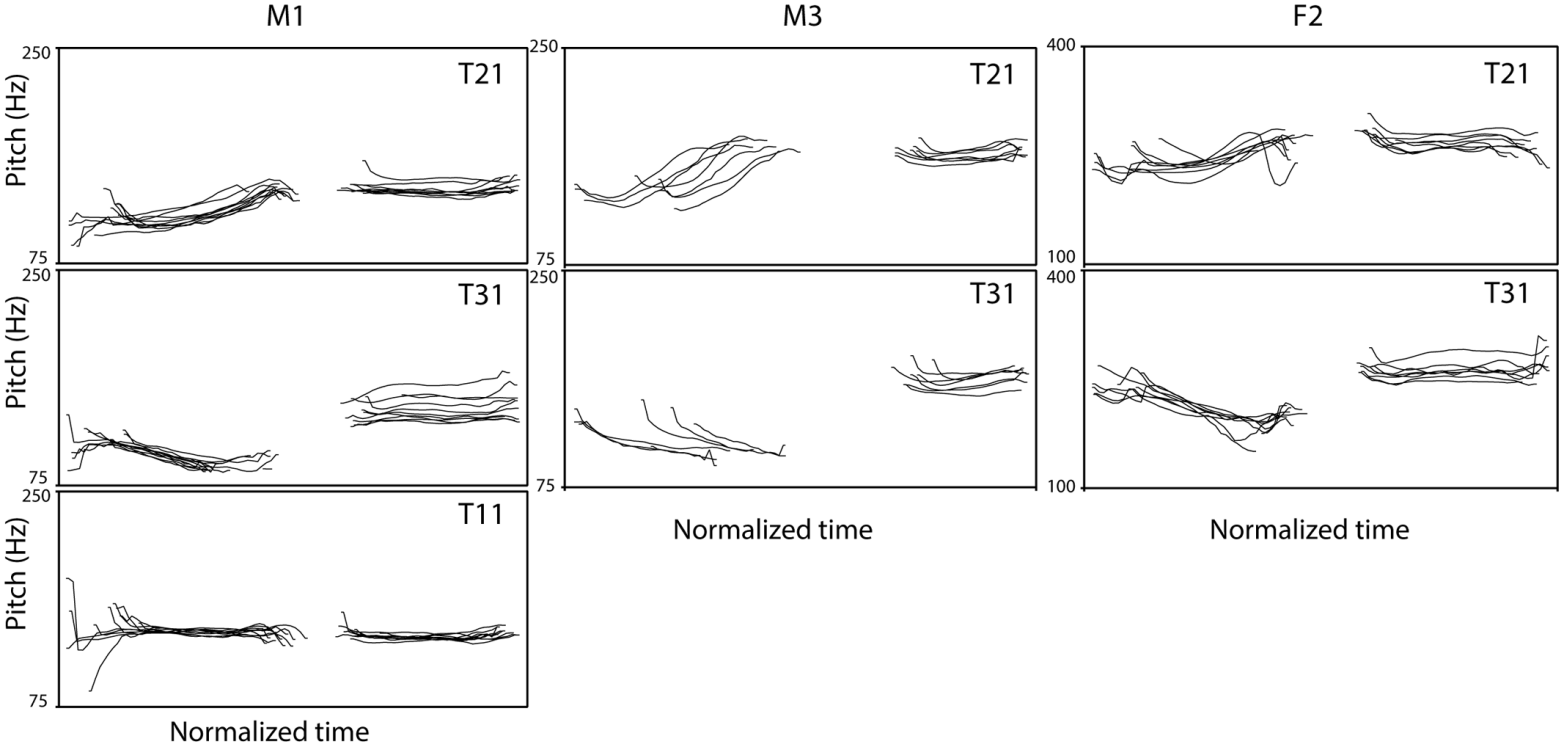
**Examples of pitch contours of bisyllabic stimuli.** Duration of tokens ranged from 878–1111 ms (M1), 830–1011 ms (M2), and 740–949 ms (F1) and these were normalized here (x-axis, Time). Tokens were aligned at the onset of the first consonant. The left column (speaker M1) presents contours of test stimuli (10 contours each), the middle column (speaker M2), and the right column (speaker F1) present examples of training stimuli (eight contours each).

***Materials*.** Two types of bisyllabic Mandarin pseudowords were used with either tone 2 on the first syllable and tone 1 on the second syllable (T21) or with a tone 3 – tone 1 sequence (T31) during training. Naturally spoken pseudowords were recorded. Five native speakers of Mandarin Chinese (two females, F1 and F2, and three males, M1–M3) recorded 41 bisyllabic pseudowords. **Figure 1** illustrates examples of the pitch contours. Note that we selected tokens that had no creaky voice in the T3 condition. The 41 words consists of 10 different first consonants (C_1_, e.g., /pasa/, /dasa/), four different vowels (V_1_, e.g., /pasa/, /pisa/), and one without C_1_, /asa/ (see **Table 2** for detail). The second syllable in all of the pseudowords was /sa/. All recordings were first low-pass filtered at 5000 Hz and average sound levels were normalized to 70 dB using Praat (Boersma and Weenink, 2008). Note that while this low-pass filtering will remove some high-frequency information that listeners could use to identify the fricative consonants in the stimuli (especially the /s/ in the second syllable), it certainly does not remove all information (/s/ can be reliably identified even after low-pass filtering at 3000 Hz; McQueen, 1991).

**Table 2:** Summary of stimuli.

				v1	Speakers
Test	Condition	Example	c_1_	(“phonetic” variability)	(“acoustic” variability)
Training	low	T21-T31/asa/	c,ch,d,f,l,m,n,p,t,zh	a	M1
Training	medium	T21-T31/asa/	c,ch,d,f,l,m,n,p,t,zh	a,e,i,u	F2, M1, M3
Training	high	T21-T31/asa/	c,ch,d,f,l,m,n,p,t,zh	a,e,i,u	F1, F2, M1, M2, M3
Identification		T11-T21-T31 /asa/	c,ch,d,f,l,m,n,p,t,zh	a	M1
Transfer	new position	T11-T12-T13 /asa/	c,ch,d,f,l,m,n,p,t,zh	a	M1
Transfer	new speaker	T11-T21-T31 /asa/	c,ch,d,f,l,m,n,p,t,zh	a	F3
Transfer	new tone	T14-T24-T34 /asa/	c,ch,d,f,l,m,n,p,t,zh	a	M1
Transfer	sentence	T11-T21-T31 /asa/	c,ch,d,f,l,m,n,p,t,zh	a	M1
Transfer	new vowel	T11-T21-T31 /üsa/	c,ch,d,f,l,m,n,p,t,zh	ü	M1

*Consonants and vowels are transcribed in Pinyin*.

**Table 3** provides example sets of training materials in each of the three variability conditions. The low-variability training condition used 1 speaker (M1) with 10 first consonants (C_1_) and one first vowel (V_1_, /a/). Two new minimal pairs with two types of C_1_ were presented each day for this group, resulting in 10 minimal pairs after the five training sessions. This set of 10 pairs is identical to those used in the pre- and post-training identification tests (see Pre- and Post- Training Identification Tests). The mid-variability training materials employed three speakers with 10 types of C_1_ and four types of V_1_. Of the five speakers, M1, M3, and F2 were used in this condition. The high-variability condition was run before the medium-variability condition, and indicated the following correct response rates by speaker: F1:0.78, F2:0.86, M1:0.84, M2:0.77, M3:0.82. The voices with the highest scores were used in the medium-variability condition (note that this choice does not artificially increase the chance of observing a difference between the medium- and high-variability conditions; on the contrary, it makes it more difficult). M1 was always presented in the first session, followed by F2 or M3 randomly in the second session and the remaining speaker in the third session, and then, for the fourth and fifth sessions, always the speaker in this pair who had not been presented in the previous session. Eight minimal pairs were presented per day, resulting in 24 minimal pairs after five training sessions for this group. The high-variability training condition used all five speakers with 10 C_1_ and 4 V_1_ conditions. Here, male speakers were presented in sessions 1, 3, 5 and female speakers were presented in sessions 2 and 4. Each day of training presented eight minimal pairs, resulting in 40 pairs after the whole training session.

**Table 3:** Example sets of training materials.

Variability		Session 1	Session 2	Session 3	Session 4	Session 5
Low	Speaker	M1	M1	M1	M1	M1
	Stimuli	pasa, casa	lasa, tasa	nasa, fasa	masa, zhasa	dasa, chasa
Medium	Speaker	M1	F2	M3	F2	M3
	Stimuli	pasa, casa, pesa, cesa, pisa, cisa, pusa, cusa	lasa, tasa, lesa, tesa, lisa, tisa, lusa, tusa	nasa, fasa, nesa, fesa, nisa, fisa, nusa, fusa	masa, zhasa, mesa, zhesa, misa, zhisa, musa, zhusa	dasa, chasa, desa, chesa, disa, chisa, dusa, chusa
High	Speaker	M2	F1	M3	F2	M1
	Stimuli	pasa, casa, pesa, cesa, pisa, cisa, pusa, cusa	lasa, tasa, lesa, tesa, lisa, tisa, lusa, tusa	nasa, fasa, nesa, fesa, nisa, fisa, nusa, fusa	masa, zhasa, mesa, zhesa, misa, zhisa, musa, zhusa	dasa, chasa, desa, chesa, disa, chisa, dusa, chusa

*Consonants and vowels are transcribed in Pinyin*.

***Procedure*.** During training, participants had to learn to associate the two types of tonal patterns (T21 and T31) with visual labels (“1,” “2,” and “3”); they did not have to learn to associate the individual stimuli with meanings. The visual labels were numbers placed on response keys and the combination of sounds and labels was counterbalanced. Three labels, rather than two, were assigned in order to accommodate the pre- and post-training identification tests, which used three categories (see Pre- and Post- Training Identification Tests). The assigned numbers were kept constant throughout the training sessions. No specific instruction with regard to sounds was provided. That is, participants did not know which syllable/position they had to pay attention to in order to give labels to sounds.

Each training session started with a brief label-learning task followed by the 2AFC task with correct/incorrect feedback on the participant’s response. During the label-learning task, participants were presented with six repetitions of an example of each category: /asa/ with either the T21 tonal pattern or the T31 pattern. These pseudowords were presented along with their visual labels (“1” and “2,” “2” and “3,” or “3” and “1”) on keys on a computer keyboard. There was an inter-stimulus interval (ISI) of 2000 ms. During the following training task, one of the stimuli was presented per trial and participants had to press a key to indicate their categorical judgments. Feedback (correct/incorrect) was given immediately after each response. The ISI was set to 1500 ms after each press of a response key. One training session consisted of five blocks of 32 trials, which took ∼15 min in total.

#### Pre- and post- training identification tests

The pre- and post-training identification tests evaluated the identification accuracy of the participants before and after each training session.

***Materials*.** The tests used minimal trios of bisyllabic pseudowords contrasting combinations of tone 1 and tone 1 (T11), tone 2 and 1 (T21), and tone 3 and 1 (T31). The double-level tone condition (T11) was added to the trained materials (T21 and T31) in order to prevent a potential ceiling effect: 3AFC is more challenging than 2AFC. However, we did not use the T11 for the training because an effect of training on two categories can be measured on tests of three categories (Sadakata and McQueen, 2013), and this allowed us to keep the duration of the training session short. During these tests, only one speaker (M1) with 10 types of C1 and 1 type of V_1_ (/a/) as well as a trio without C_1_ were used, resulting in 11 minimal trios in total. This set is identical to the low-variability training materials (except for the additional T11 sequences in the tests).

***Procedure*.** Each identification test started with a brief label-learning task followed by a 3AFC task without any feedback. During the label-learning task, participants were presented with six repetitions of an example of each of the three categories (/asa/ with T11, T21, and T31). Each pseudoword was presented along with the three visual number labels (“1,” “2,” and “3”) each associated with a labeled key on the keyboard, with an ISI of 2000 ms. During the 3AFC task, one of the CVCV word was presented per trial and participants had to press the key to indicate their categorical judgments.

#### Transfer test

The transfer test used the same task and procedure as the pre- and post-training identification tests. The following five new types of minimal trios of stimuli were tested: (1) new position, in which the order of tones was reversed, (2) new speaker, spoken by a third female speaker (F3), (3) new tone, in which the critical (first) tone stayed the same but was combined with a new second tone, (4) new vowel, using the vowel /ü/ (/püsa/) instead of /a/, (5) sentence, in which the critical words were embedded in a sentence context: “*zhe(T4) zhi(T1) xxx shi(T4) wo(T3) de*” (‘this xxx is mine’). First, all minimal trios were presented six times as examples together with their correct visual numerical labels. Then, in the main part of the transfer test, participants heard the stimuli and were asked to remember the types of sequence and their corresponding visual labels. As in the pre- and post-training identification tests and the training, participants were not instructed which syllable/position they had to pay attention to. The transfer test consisted of 450 trials: 90 trials per 5 transfer conditions, blocked by condition.

#### Categorization of synthesized stimuli

Categorization of synthesized tonal continua (a speech continuum between tone 2 and tone 3, and an analog non-speech continuum) was administered on days 1 and 5.

**FIGURE 2:**
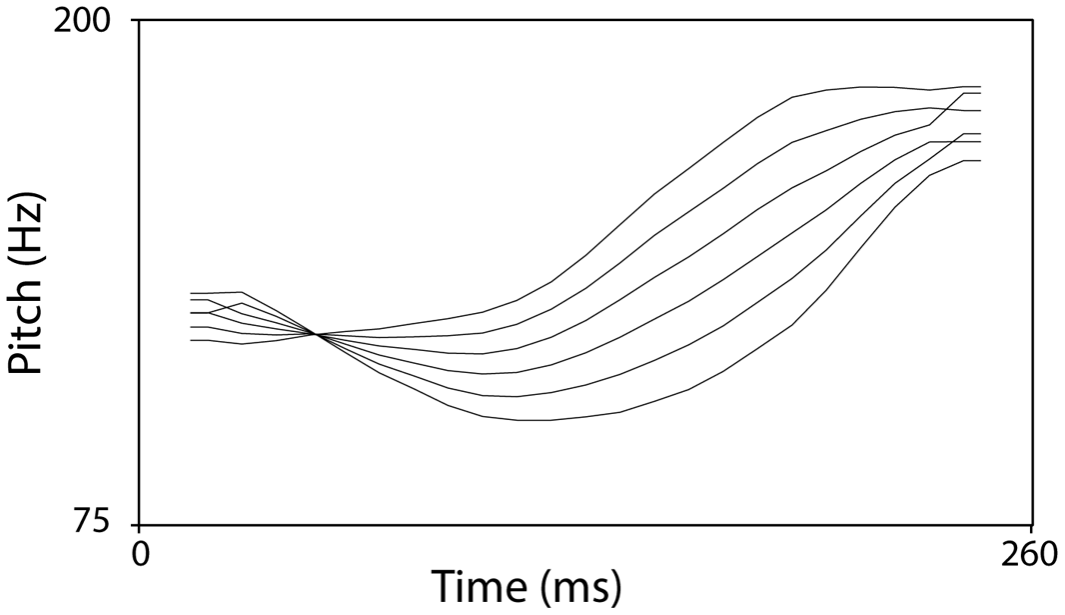
**The pitch continuum from T2 to T3**.

***Materials*.** The two continua, speech and non-speech, consisted of six steps from tone 2 to tone 3 (step 1: prototypical tone 2, step 6: prototypical tone 3). They were created by linearly interpolating between the endpoint prototypes using the PSOLA method in Praat (Boersma and Weenink, 2008). **Figure 2** shows the contour patterns of these stimuli. For the speech materials, the original sounds of monosyllable /a/ with tone 2 and tone 3 utterances were recorded by M1. The pitch patterns of these tone contours were extracted and the duration of both the tone 2 and the tone 3 segments were normalized to 260 ms. Onsets and offsets of each element were ramped (5 ms) using a cosine filter in order to avoid clipping or discontinuities. In total, there were six speech stimuli. The non-speech continuum was created by applying the contours of the six steps of the speech continuum to a sine tone.

***Procedure*.** The speech and non-speech categorization tests followed the same procedure. The experiment started with a label-learning task followed by three blocks of an identification task. During the label-learning task, participants were presented with examples of two categories (tone 2 and tone 3) six times each. Each category was presented along with its corresponding visual numerical label with an ISI of 2000 ms. During the categorization task, an auditory stimulus was presented and participants indicated their identification judgments. Each test included two blocks that each consisted of 60 trials, and lasted ∼6 min.

#### Discrimination of synthesized stimuli

The synthesized material was also used to test discrimination sensitivity. The speech and non-speech discrimination tests followed the same procedure. They employed a speeded 4I2AFC task. We opted for this task rather than the traditional 2AFC discrimination task because the former depends less on the categorical level of processing and more on lower-level acoustic-phonetic processing (Gerrits and Schouten, 2004) and thus was likely to provide complementary information to the categorization test (i.e., an indication of sensitivity to low-level acoustic differences). Each trial consisted of presentation of four stimuli. Either the second or the third stimulus of the four was a deviant. Participants were asked to indicate the position of that deviant by pressing button “2” or “3.” The probability of the deviant appearing in each position was set to 0.5. Tone 2 stimuli served as standards. Deviant stimuli were all the other sounds of the continuum. The ISI was set to 500 ms. There were two blocks, one block each for the speech and non-speech stimuli, each consisting of 50 trials. This test lasted ∼9 min. Participants completed at least one practice session of six trials using a dummy word pair (/put/-/pet/ or a non-speech analog) before the main session. No feedback was provided on task performance.

### APPARATUS

Exactly the same apparatus was used as in Sadakata and McQueen (2013). A linear PCM recorder (Sony PCM – D1) was used to record all the stimuli (sampling rate of 96 kHz). For the running of the experiment, a DELL notebook computer with an IntellCoreDuo processer (4 GB RAM) was used. The participants responded on the computer keyboard. Sound was presented through Sony MDR-7506 headphones. Visual instructions and stimuli were presented via a 15.4-inch TFT screen. Average sound pressure level (SPL) of the headphones was adjusted to ∼69 dB. The experiment was programmed using Presentation software (version 14.3, Neurobehavioral Systems).

## RESULTS

The primary analyses are those examining the interaction between type of training and individuals’ aptitude in tonal perception. First, however, we will describe the overall analyses examining the effect of type of training materials on perceptual learning for all participants together. Prior to all analyses, outliers were identified as responses with a reaction time longer than 3 standard deviations from the grand mean for each task. About 1.7% of all responses were excluded.

### TRAINING

**FIGURE 3:**
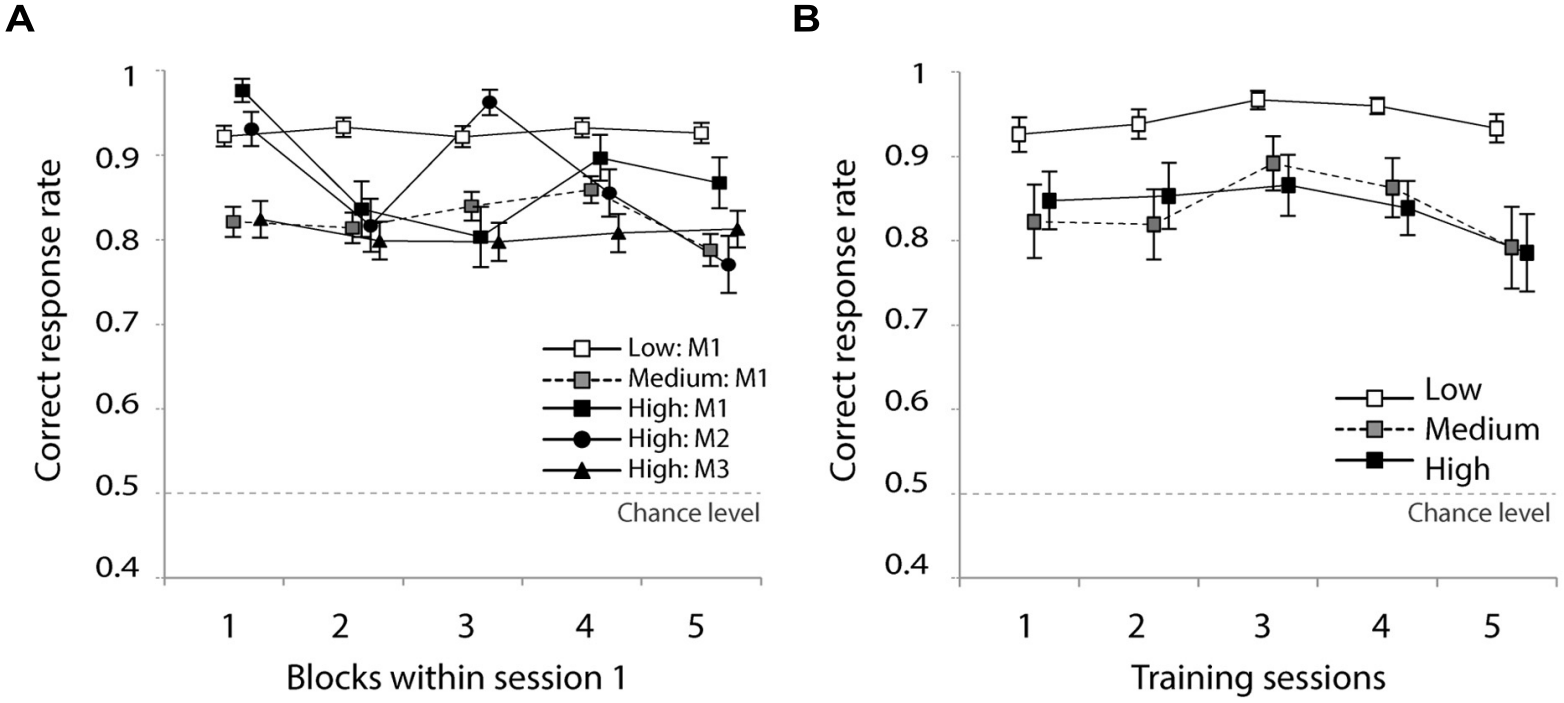
**(A)** Correct response rate, five blocks in the first training session and **(B)** over the five training sessions. Error bars indicate standard errors.

**Figure 3A** presents the correct response rate in the first training session for the five types of training materials. A two-way analysis of variance (ANOVA) with block (continuous variable) and training material (low-M1, medium-M1, high-M1, high-M2, high-M3) as independent variables and participants as random variable revealed a significant main effect of block [*F*(1,171) = 4.1, *p* < 0.05], indicating that the correct response rate decreased slightly over the course of the training session. This is likely to be a fatigue effect. The analysis found neither a significant effect of training material [*F*(4,171) < 1, n.s.] nor an interaction [*F*(4,171) < 1, n.s.]. This means that the different training materials did not result in differences during the first training session.

**Figure 3B** shows the correct response rate in the five training sessions for the low, medium, and high groups. An ANOVA with group (high-/medium-/low-variability) and training session (continuous variable) as independent variables and participants as random variable indicated a significant main effect of group [*F*(2,42) = 4.8, *p* < 0.05]. The main effect of session and the interaction were not significant [session; *F*(1,170) = 1.2, n.s., interaction; *F*(8,140) < 1, n.s.]. A follow-up multiple comparison (Student’s *t*-test) indicated that the correct response rate of the low group was significantly higher than that of the high group (*p* < 0.05).

### IDENTIFICATION TEST

**FIGURE 4:**
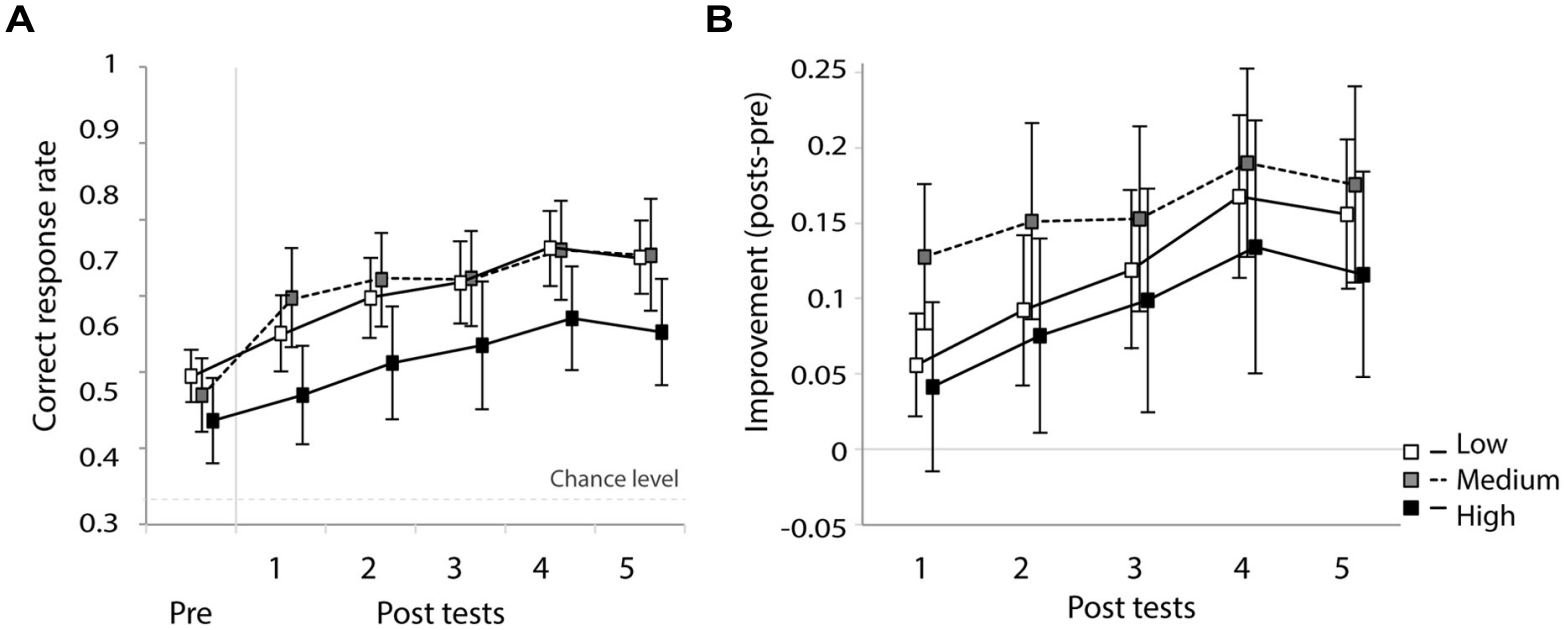
**(A)** Correct response rate in the first pre-test and five post-tests and **(B)** improvement rate relative to the first pre-test. Error bars indicate standard errors.

**Figure 4A** presents the correct response rate of the first day pre-test followed by five post-tests for the three training groups. A one-way ANOVA confirmed that there was no significant difference between the groups’ correct response rates in the first day pre-test [*F*(2,41) < 1, n.s.]. This confirms that there was no group difference with regard to performance accuracy prior to the training sessions. **Figure 4B** presents improvements of correct response rates for five sessions. The correct response rate of the first day pre-test is subtracted from the five post-tests. A two-way ANOVA with training sessions (continuous variable) and group (high-/medium-/low-variability) as independent variables and participants as random variable on the improvement rates revealed a strong and significant main effect of training session [*F*(1,171) = 51.0, *p* < 0.001], with a tendency to improve performance over the course of the five sessions. Neither the main effect of group [*F*(2,215) < 1, n.s.] nor the interaction [*F*(2,171) = 1.2, n.s.] was significant. This indicates that all groups improved their identification accuracy during the five training sessions and, although the medium group seemed to show the most improvement, there was no significant group difference.

### TRANSFER TEST

**FIGURE 5:**
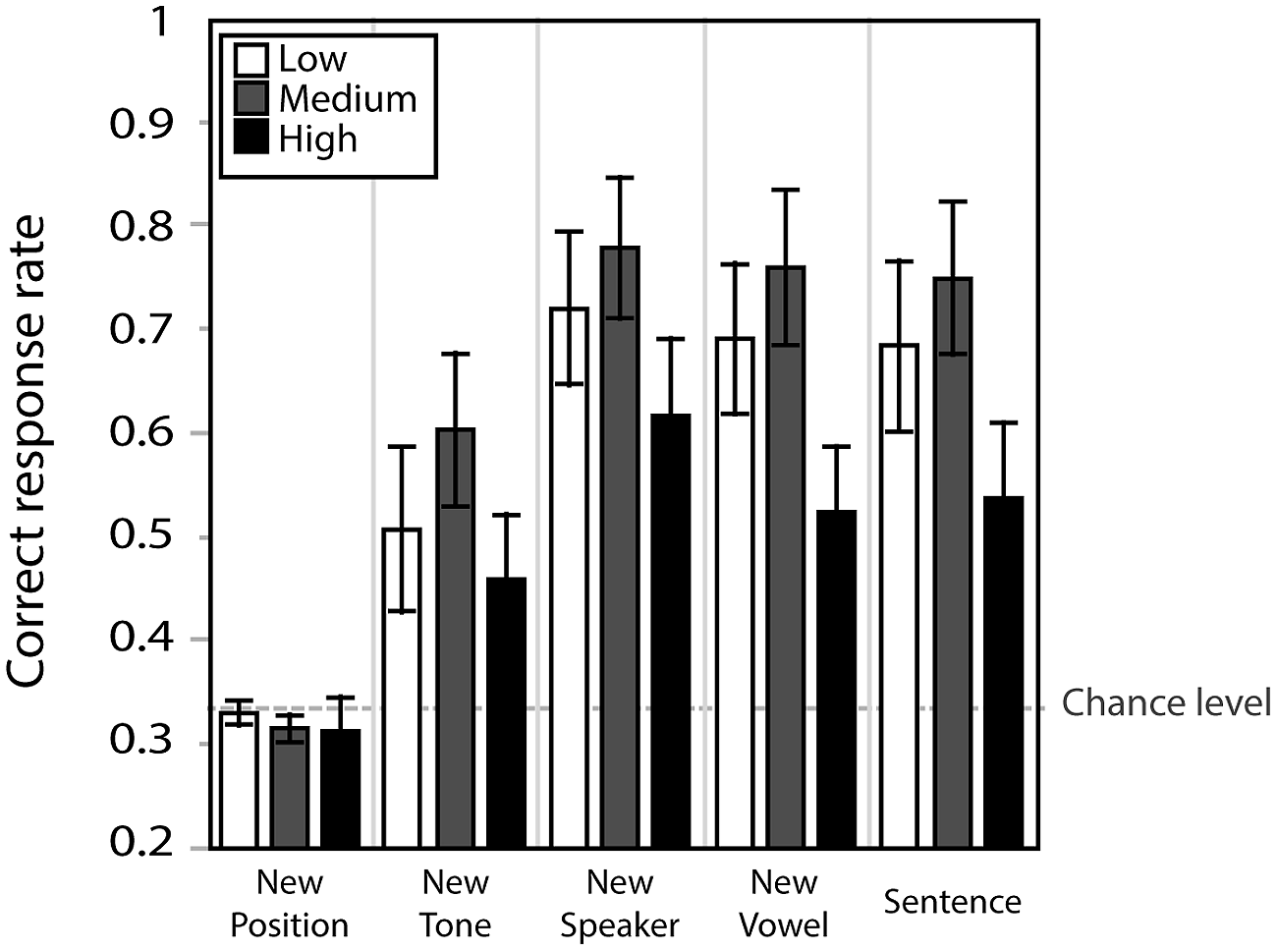
**Correct response rate in the five transfer conditions.** Error bars indicate standard errors.

**Figure 5** presents correct response rates for the five transfer conditions. A repeated measure ANOVA with group (high-/medium-/low-variability) as between subject independent variable and transfer condition (five conditions) as within-subject independent variable indicated no significant effect of group [*F*(2,39) = 2.2, n.s.], an effect of condition [*F*(4,156) = 49.8, ε_GG_0.581, *p* < 0.001] and no significant interaction [*F*(8,156) = 1.9, ε_GG_0.581, n.s.]. Simple effect analyses indicated that correct response rates in the new position condition, the one which reversed the tones of two syllables (e.g., T12 instead of T21), was significantly lower than the other conditions (*p* < 0.001). In fact, 95% confidence intervals of correct response rate in this condition overlapped with chance level (low: 0.12–0.35, medium: 0.14–0.34, variable 0.14–0.39). The perceptual learning that was demonstrated in the previous results was therefore specific to the first syllable of the stimuli. The new tone condition, combining the critical tones (T2 and T3) with a new context tone (e.g., T24 instead of T21), was the second hardest condition. Identification accuracy of this condition was significantly lower than the other three conditions (*p* < 0.001). We performed a further analysis to check if any specific tone was contributing to lowering the task performance here. A two-way repeated-measure ANOVA with tone categories (T14, T24, T34) as a within-subject factor and group (high-/medium-/low-variability) as a between-subject factor indicated no significant main effects [tone categories: *F*(2,38) < 1, n.s., group: *F*(2,39) < 1, n.s.]. This suggests that the decrease in task accuracy in this condition was not due to one specific tone category, but due instead to altering the context from T1 to T4. However, the fact that participants performed better in the new tone than the new position condition suggests that they learned to hear pitch contours of the first syllable to some extent independently of the following context.

### CATEGORIZATION OF SYNTHESIZED CONTINUA

**FIGURE 6:**
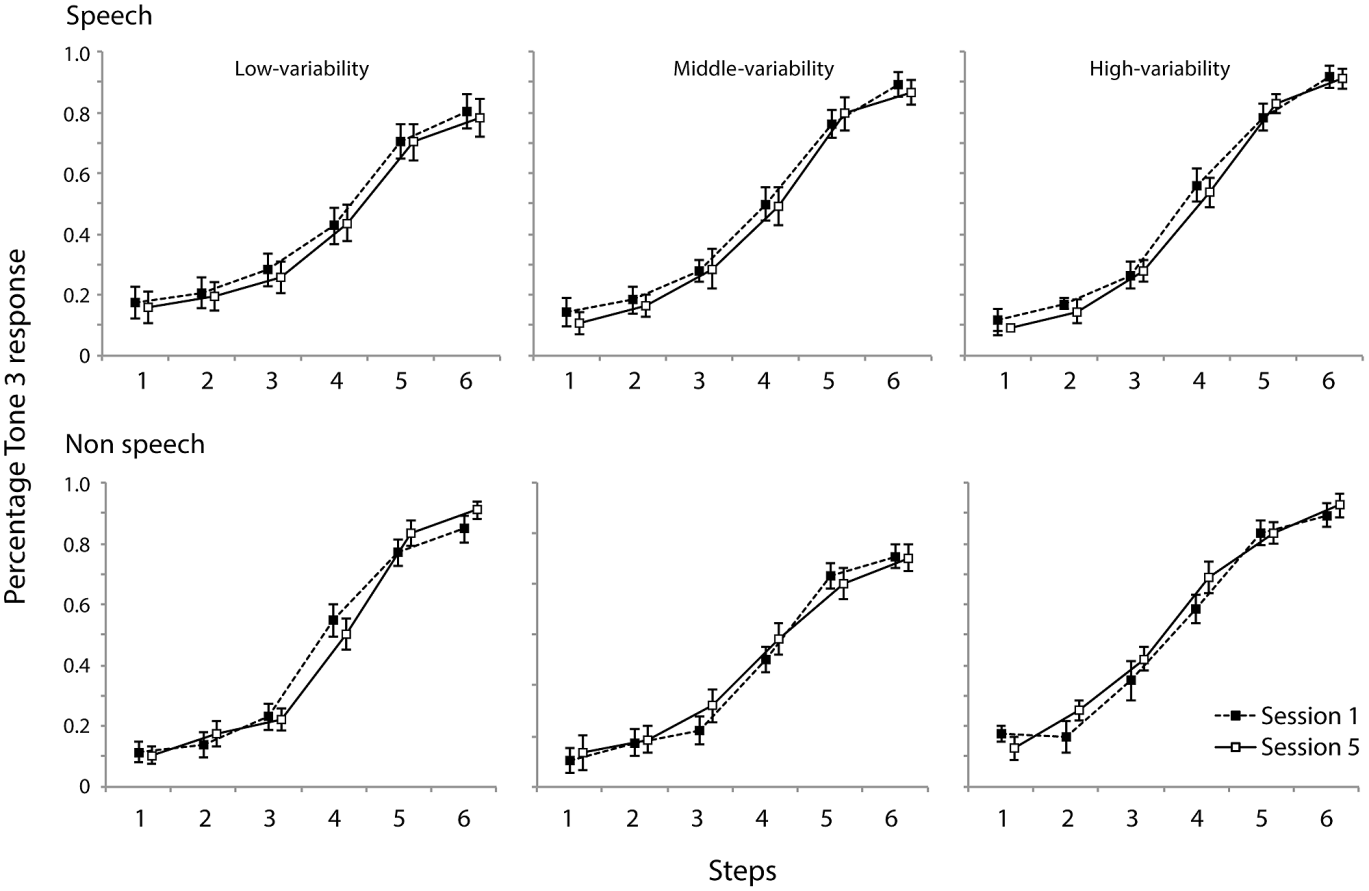
**Categorization of synthesized speech and non-speech materials for all three groups**.

**Figure 6** shows percentage of “Tone 3” responses as a function of contour continua for days 1 and 5, respectively for the speech and non-speech stimuli for three training groups.

The slope of each individual’s categorization function was estimated using the curve logistic function in SPSS (ver.20). The steeper the functions are, the smaller the coefficients. Coefficients larger than 1.2 represents poor estimates of functions (Joanisse et al., 2000); three values were discarded from the analysis for this reason. The mean slope coefficients for each group in the speech and non-speech conditions are summarized in **Table 4**. Two-way mixed-model ANOVAs with group (high-/medium-/low-variability) and day (day 1/day 5) as within-subject factors were carried out separately for the speech and non-speech data. Neither analysis indicated a main effect of group [speech: *F*(2,40) < 1, n.s., non-speech: *F*(2,40) < 1, n.s.] or day [speech: *F*(2,40) < 1, n.s., non-speech: *F*(2,40) < 1, n.s.]. This indicates that the training did not influence categorization performance.

**Table 4:** Estimated slope coefficients for the three training types.

Variability in training materials	Speech		Non-speech	
	Day 1	Day 5	Day 1	Day 5
Low	0.391 (0.10)	0.360 (0.10)	0.221 (0.07)	0.270 (0.09)
Medium	0.253 (0.07)	0.260 (0.08)	0.211 (0.07)	0.195 (0.07)
High	0.200 (0.05)	0.231 (0.06)	0.200 (0.07)	0.166 (0.08)

*Numbers in brackets are standard errors*.

### DISCRIMINATION OF SYNTHESIZED CONTINUA

**FIGURE 7:**
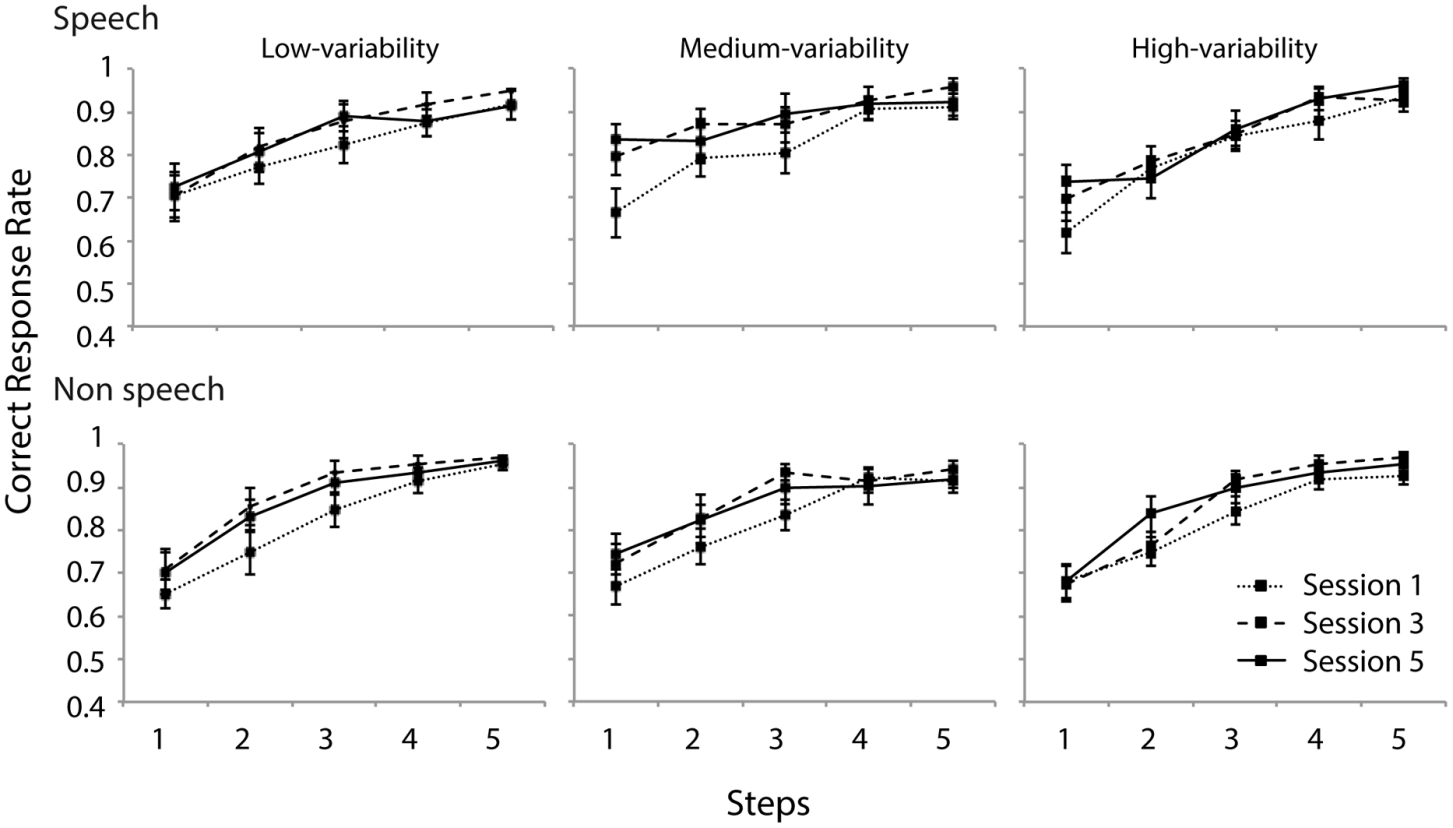
**Discrimination accuracy of speech and non-speech stimuli for all three groups.** Contour difference increases as a function of five contour steps. Error bars indicate standard errors.

**Figure 7** presents the discrimination data (proportion of correct responses). Two mixed-model ANOVAs were performed with group (low-/medium-/high-variability) as between-subject factor and day (day 1/3/5) as within-subject factor. For both analyses, no effect of group was observed [speech: *F*(2,37) < 1, n.s., non-speech: *F*(2,37) < 1, n.s.]. Also, no interaction effect with regard to training group was observed. The results for the speech stimuli indicated main effects of day [*F*(2,74) = 6.1, ε_GG_ = 0.655, *p* < 0.01] and step [*F*(4,148) = 67, ε_GG_ = 0.510, *p* < 0.0001] and an interaction between them [*F*(8,296) = 3.5, ε_GG_ = 0.63, *p* < 0.001]. Further simple analyses indicated that the correct response rates on days 3 and 5 were significantly higher than on day 1 for the medium-variability group (*p* < 0.05), and that on day 5 was significantly higher than that on day 1 for the high variable group (*p* < 0.05). Although the ANOVA did not reveal an overall effect of training type, these two groups appeared to improve their discrimination sensitivity to the smallest contour contrast.

Similar to the speech condition, the analysis of non-speech data revealed main effects of day [*F*(2,74) = 8.2, ε_GG_ = 0.777, *p* < 0.001] and step [*F*(4,148) = 97.1, ε_GG_ = 0.605, *p* < 0.0001]. No interaction between them was present [*F*(8,74) = 1.9, ε_GG_ = 0.654, n.s.]. Further simple effect analyses revealed that the correct response rates of days 3 and 5 were significantly higher than that of day 1. For the non-speech stimuli too, larger contour contrast resulted in higher discrimination accuracy. Differences between steps were all significantly different (*p* < 0.01) except for the last two steps (4 and 5).

### INDIVIDUAL DIFFERENCES

**FIGURE 8:**
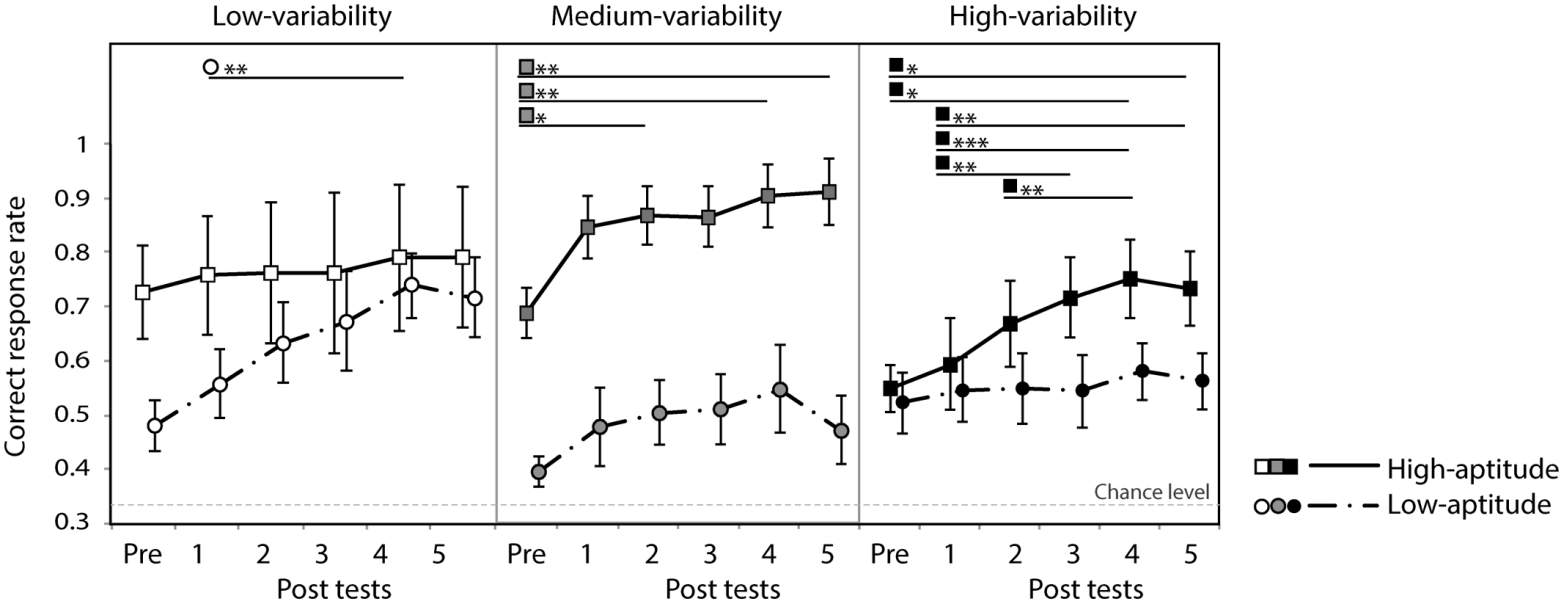
**Correct response rate in the first pre-test and five post-tests for low- and high-aptitude perceivers in the three variability conditions.** Error bars indicate standard errors. *p < 0.05, **p < 0.01, ***p < 0.001.

Participants were grouped into two classes (high and low perceptual aptitude groups) according to their identification slope coefficients of the day 1 and day 5 speech categorization data. For the three cases of missing coefficient due to poor fit, only one coefficient was available (either day 1 or day 5) and these were used to form groups. Steep and shallow categorization slopes were classified using a cutoff of value of 0.2: participants were categorized as low-aptitude perceivers if either the day 1 or day 5 coefficients exceeded the cutoff value. This resulted in the following division in each training group (number of participants in high/low-aptitude groups): low (8/7), medium (6/9), and high (7/8). **Figure 8** shows the correct response rate of the identification tests (the first pre-test and the five post-tests) for each training type in the two aptitude groups. A three-way repeated measure ANOVA was performed with session as within-subject factor and training group (low-/medium-/high-variability) and aptitude (high/low) as between-subject factors. There was a strong main effect of session [*F*(5,170) = 16.8, *p* < 0.0001] and of aptitude [*F*(1,34) = 11.3, *p* < 0.01]. No effect of training group was found [*F*(2,34) < 1, n.s.] but there was a significant three-way interaction of these factors [*F*(10,170) = 2.2, *p* = 0.022]. Significant simple effects are indicated in **Figure 8**. In general, the low-aptitude perceivers tended to improve only in the low-variability condition while the high-aptitude perceivers improved in the medium- and high-variability conditions.

**FIGURE 9:**
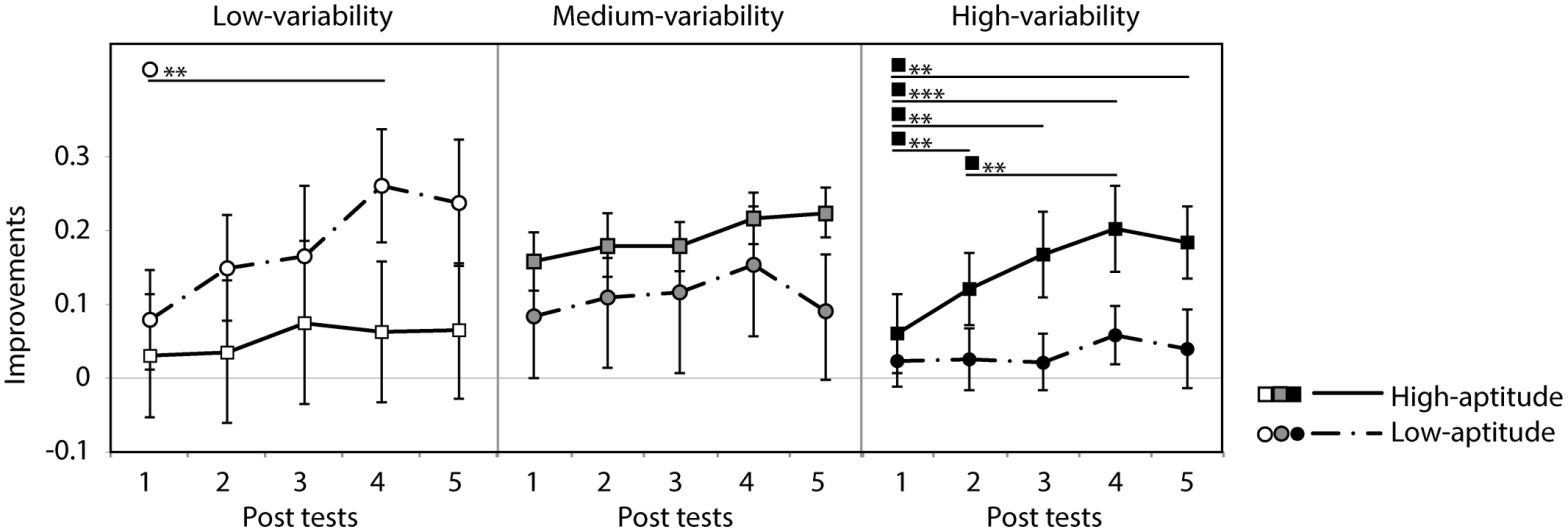
**Improvement rate in the five post-tests for low- and high-aptitude perceivers in the three variability conditions.** Error bars indicate standard errors. **p < 0.01, ***p < 0.001.

As can be seen in **Figure 8**, however, the six groups differed at baseline (i.e., in their pre-test identification accuracy). These differences are most likely to be spurious, reflecting in part the random assignment of participants to variability condition and in part the *post hoc* split based on the participants’ categorization data. These baseline differences, however, make it hard to interpret the three-way interaction found in the analysis of the absolute identification data. In order to control for these baseline differences and hence to test directly whether the participants in the different groups learned differentially, improvement rates were computed (improvements in correct response rate over the course of the five post-tests relative to the pre-test score). **Figure 9** presents these data. The improvement rates were submitted to a three-way repeated measure ANOVA with session as within-subject factor and training group (low-/medium-/high-variability) and aptitude (high/low) as between-subject factors. There was a strong main effect of session [*F*(4,136) = 13.1, *p* < 0.0001]. There were no main effects of aptitude [*F*(1,34) = 1.0, n.s.] and training group [*F*(2,34 < 1, n.s.]. However, there was again a significant three-way interaction of these factors [*F*(8,136) = 3.0, *p* < 0.05]. Significant simple effects are indicated in **Figure 9**. The pattern in the previous analysis is clearer after baseline differences have been controlled: significant improvements were observed for low-aptitude perceivers in the low-variability condition, but for the high-aptitude perceivers in the high-variability condition.

The main finding that low-aptitude perceivers improved more in the low-variability condition than in the mid- and high-variability conditions and the high-aptitude perceivers improved most in the high-variability condition could potentially be explained by differences in aptitude across the variability conditions. In order to test this possibility, a two-way ANOVA with training group (low-/medium-/high-variability) and aptitude (high/low) on the average slope coefficients of day 1 and day 5 speech data was performed. It indicated a significant main effect of aptitude [*F*(1,39) = 80.8, *p* < 0.0001] but not of training group [*F*(2,39) = 2.2, n.s.], with a significant interaction between these factors [*F*(2,39) = 3.4, *p* < 0.05]. Multiple comparison (Student’s *t*-test) analysis indicated that the slope coefficients of the low-aptitude perceivers did differ among training conditions. Critically, however, the coefficients of these participants in the high-variability condition were significantly smaller than those of these participants in the other two conditions (*p* < 0.05). That is, the low-aptitude perceivers in the high-variability condition – not those in the low-variability condition (i.e., those who showed the greatest improvements over training) – tended to be better in categorizing sounds than those in the other two conditions. Furthermore, there were no differences in the slope coefficients of the high-aptitude perceivers across conditions. These analyses therefore suggest that our main findings cannot be explained by differences in individuals’ aptitude within the aptitude groups across variability conditions.

**FIGURE 10:**
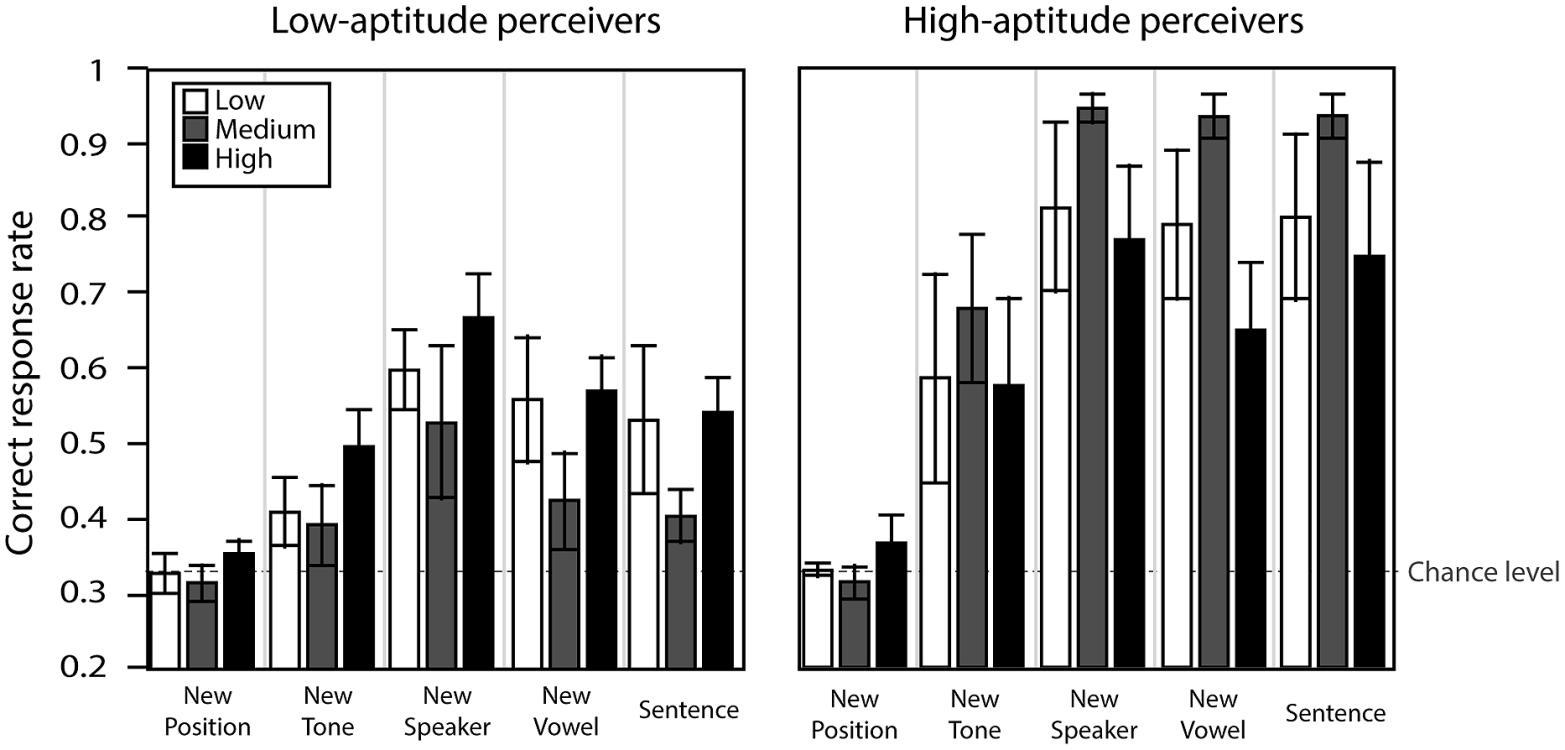
**Correct response rate in the transfer condition for low- and high-aptitude perceivers in the three variability conditions.** Error bars indicate standard errors.

We further studied the interaction of aptitude and training type on generalization ability. **Figure 10** presents the correct response rate for the five transfer conditions for both aptitudes and for all training conditions. A three-way repeated measure ANOVA with transfer condition as within-subject factor and variability training group and aptitude group as between-subject factors was performed on the correct response rate in each of the transfer tests. The analysis indicated a significant main effect of transfer condition [*F*(4,132) = 48.3, ε_GG_ = 0.664, *p* < 0.0001] and aptitude [*F*(1,32) = 20.5, *p* < 0.0001], with a significant interaction between condition and aptitude [*F*(4,140) = 7.8, *p* < 0.0001]. There was no effect of variability training group and no interactions involving this factor. Further simple effect analysis confirmed that the high-aptitude group demonstrated higher correct responses than the low-aptitude group in all conditions except for the new position condition, which was overall the hardest one.

## GENERAL DISCUSSION

The current study investigated the effect of the high-variability training method on non-native lexical tone learning by native Dutch listeners. In general, all groups improved their identification accuracy of bisyllabic pseudowords with naturally spoken Mandarin tone contours over the course of five sessions, indicating that it is possible for Dutch native listeners to learn to perceive this type of contrast, at least to some extent. However, unlike some previous studies that demonstrated clear across-the-board benefits of high-variability training (Logan et al., 1991; Wang et al., 1999; Hirata et al., 2007; Sadakata and McQueen, 2013), no apparent overall benefit of high-variability was observed here. This replicates other previous studies where high-variability training did not necessarily enhance perceptual learning (Kingston, 2003; Wade et al., 2004; Perrachione et al., 2011). It is particularly interesting to note that, while using the identical protocol, the current results deviated considerably from those of our previous study (Sadakata and McQueen, 2013). Notably, more detailed analyses with regard to individuals’ perceptual aptitude revealed an interaction: increased variability hindered perceptual learning in low-aptitude perceivers while it enhanced perceptual learning in high-aptitude perceivers. We have thus demonstrated that this interaction, previously demonstrated in a word-meaning learning task with simple materials (monosyllabic and synthetic, Perrachione et al., 2011), also applies to the learning of categorical labels for multi-syllabic words with naturally spoken lexical tones.

This study used a procedure closely matched with Sadakata and McQueen (2013) in order to make a comparison of the effects of different training methods when learning phonetic contrasts that are very different in nature (Mandarin tones vs. Japanese geminate consonants). One may be concerned, however, that the analysis methods in the two studies with regard to individual differences were different [linear regression in Sadakata and McQueen (2013)]. We therefore performed the same individual difference analysis on the geminate data as was done here in order to confirm if the results are indeed different. The same grouping criteria were applied, based on the same type of data: slope coefficients for the categorization of synthetic speech stimuli. Averaged slope coefficients of three types of speech stimuli (geminate consonants with three preceding vowel durations, see the original study for detail) were computed for each of day 1 and day 5. If any of these coefficients exceeded the cutoff value of 0.2, participants were classified into the low-aptitude group. These criteria resulted in the following divisions (number of participants in high/low-aptitude groups): low (9/6) and high (9/6). Three-way mixed-design ANOVAs were performed, one using correct response rate of the identification tests (six sessions) and another using improvements of correct response rates (five sessions relative to pre-test) as within-subject factors. Between-subject factors were training group (low-/high-variability) and aptitude (high/low). The results indicated the absence of interaction effects with regard to aptitude. The ANOVA on the absolute correct response rate revealed an effect of session [*F*(5,130) = 36.3, ε_GG_ = 0.670, *p* < 0.001] but not of group [*F*(1,26) = 0.6, n.s.] and aptitude [*F*(1,26) = 0.3.2, n.s.], and the only significant interaction was between session and group [*F*(5,130) = 4.4, *p* < 0.001]. The ANOVA with improvement rate revealed effects of session [*F*(4,104) = 25.5, *p* < 0.001] and group [*F*(1,26) = 9.5, *p* < 0.01] but not of aptitude [*F*(1,26 = 0.071, n.s.), and there were no significant interactions. These findings clearly indicate that, in contrast to the current study, there was no modulation of the effects of variability by perceptual aptitude on the learning of the Japanese geminate/singleton consonant contrast.

The choice of the current experimental design, however, restricted the generalizability of our results when comparing with other training studies. The current training material contrast (T21 and T31) is one of the most challenging bisyllabic tone combinations for Dutch native listeners, at least according to a pilot study we carried out prior to this study. This contrast may be difficult because T31 includes only a partial third tone. The partial tone is due to a tone sandhi rule which means that the normal final upward pitch movement seen in a T3 in isolation is omitted in the T1 context, such that the pitch contour moves more smoothly into the following T1. This may explain why no transfer was observed in the categorization and discrimination tests (those with the synthesized continua) and in the new position condition in the transfer test (i.e., the contrast between T12 and T13). In both these cases the materials consisted of full rather than partial T3 contours. This, in turn, indicates that our identification training protocol mostly improved skills to perceive specific pitch contour patterns. Alternatively, the failure of the position transfer test could have been due to the ambiguity in our instruction about where to listen to in this condition: participants may have been expecting to hear the critical difference at the first syllable position instead of the second because they were not told which tone they should pay attention to.

Interestingly, both high- and low-aptitude perceivers showed no transfer in the new position condition, while high-aptitude perceivers outperformed low-aptitude perceivers on all other transfer conditions (i.e., there was no interaction of variability condition and aptitude in the transfer test data). One might have expected that if variability and aptitude interact in performance improvements on the trained stimuli, there would also have been a similar interaction in the transfer tests. But this would only follow if learning had led to the formation of fully abstract representations of T2 and T3. If, in contrast, as we have just suggested, learning was about the specific tonal patterns heard during training, then one would predict that there would be poor transfer overall (as observed in the new position and new tone conditions), and that performance on the transfer tests would reflect perceptual aptitude. This is exactly what was observed: the high-aptitude perceivers performed better than the low-aptitude perceivers in all transfer tests except the new position condition (where all participants were at chance). In other words, the transfer data simply reflect the split in perceptual aptitude that was based on initial categorization performance. This argument thus also suggests that, at least for the participants in the current study, the training – with only bisyllabic sequences and a single tonal context (only T1) – did not lead to the formation of fully abstract categories for T2 and T3.

Three types of variability were included in our training stimuli: across conditions, there was variability in the number of speakers (“acoustic variability”), the number of words (“phonetic variability”), and the number of repetitions of different individual tokens. Unlike other high-variability training paradigms that intermixed speakers within the same session (e.g., Perrachione et al., 2011; Cooper and Wang, 2013), we presented one speaker per day to the listeners in our variable conditions, as in Sadakata and McQueen (2013), in order to restrict the amount of trial-by-trial acoustic variability given the complex nature of our bisyllabic stimuli. Nevertheless, overall, we manipulated the three types of variability factor (acoustic, phonetic, and repetition) at the same time in order to maximize the contrast between groups with regard to their experience of variability. However, this makes it challenging to identify which type of variability was responsible for the reported interaction effect. It is possible that, as in Perrachione et al. (2011), phonetic variability and differences in token repetition across conditions may have played the primary role. In particular, increased repetition of tokens in the low-variability condition relative to the high-variability condition may have benefitted the low-aptitude listeners. But it is not possible to verify this given our study design. Future studies will need to address this issue.

The medium-variability condition was added in order to gain more fine-grained insights into the interaction of variability and listener aptitude. The three speakers used in this condition were the most intelligible ones in the high-variability training condition. This means that the degree of speaker intelligibility across our training groups did not increase linearly. More generally, speaker intelligibility was not fully controlled across all three conditions (as could be achieved if speakers were fully rotated across conditions, as in Perrachione et al., 2011). Future research is thus required to establish whether the present pattern of results is weaker (or stronger) when speaker intelligibility is fully controlled. Nevertheless, it appears unlikely that the current pattern of results could be caused solely by intelligibility differences. First, note that speaker M1, whose recordings were used in all three conditions, was the speaker with the second-highest accuracy score in the high-variability condition. If the high-aptitude participants’ better performance in the high-variability condition was due only to the addition of other speakers in that condition who were more intelligible, accuracy on M1 should have been relatively lower. Second, and more generally, there seems to be no simple explanation for the aptitude by variability interaction: it seems unlikely, for example, that the speakers which high-aptitude perceivers would find more intelligible would be less intelligible for low-aptitude perceivers.

What then comprises the individuals’ perceptual aptitude? In previous studies, the Pitch-Contour-Perception-Test (PCPT) was used to assess each individual’s aptitude (Wong and Perrachione, 2007; Perrachione et al., 2011; Ingvalson et al., 2013). This test measures whether someone can perform auditory identification of three pitch contours (rising, falling, and level) superimposed on a single syllable. Our study used the steepness of categorization functions as an indicator of aptitude. In essence, both methods point to the same ability, namely, to form a perceptual group based on pitch contour information. We suggest that at least two functions support this ability. One is sensitivity to pitch information at early perceptual level, such as one measured by pitch discrimination tests (e.g., Demany, 1985) and by the frequency following response (FFR) observed at the brainstem (e.g., Wong et al., 2007b). Having access to a more faithful coding of pitch patterns ought to be helpful when perceptual learning relies on this acoustic feature. There are considerable differences among individuals with regard to this ability based on their sound experiences, such as L1 and musical training (Krishnan et al., 2005; Wong et al., 2007b; Bidelman et al., 2011; Sadakata and Sekiyama, 2011).

The other important function that correlates with individuals’ aptitude in non-lexical tone learning is flexibility in remapping the weightings of perceptual cues. Direction of pitch contour and height are main cues of pitch information in speech. How one’s native language uses pitch information, that is, for either lexical tone contrasts (in a tonal language) or intonation contrasts (in a non-tonal language), results in different ways to weight these cues (Francis et al., 2008): native speakers of tonal languages put more weight on pitch direction whereas those of non-tonal languages put more weight on pitch height (Guion and Pederson, 2007; Kaan et al., 2007). A shift in perceptual cue weighting from pitch height to pitch direction therefore needs to occur when native speakers of non-tonal languages learn to perceive lexical tones. How flexibly and quickly one can achieve this shift seems to be important part of the individual’s aptitude. Chandrasekaran et al. (2010) show, for example, that better learners of lexical tones tend to attend more to pitch direction than to pitch height, while poorer learners tend to attend more to pitch height.

For native listeners of Dutch, this remapping of the use of pitch information is crucial when learning lexical tone contrasts. But remapping is not required when they learn to perceive Japanese geminate/singleton consonants (Sadakata and McQueen, 2013). One of the important features that determine the Japanese geminate/singleton contrast is timing: duration of consonants and their surrounding phonemes largely characterizes this contrast (Han, 1994; Kingston et al., 2009; Amano and Hirata, 2010, but see also Idemaru and Guion, 2008). Although timing is used to contrast different types of phonemes in speech, such as VOT (Brandmeyer et al., 2012), vowels (Ylinen et al., 2005) and consonants (e.g., the geminate/singleton contrast), learning a non-native contrast based on timing seems to require a much simpler adjustment compared to learning of pitch contrasts. This difference may explain why there was an overall benefit of high-variability training in the geminate study, and instead the aptitude by variability interaction in the present study, in spite of the use of identical training protocols. In learning of lexical tones, the extreme remapping of pitch function may have served as an extra source of individual difference, which interacted more with the effect of high-variability training methods than in learning the Japanese geminate/singleton contrast. It would be interesting to look further into the interaction effect between individual differences and training variability by testing other contrasts than tones and/or by testing tones with individuals whose mother tongue is at the border between tonal and non-tonal languages (e.g., Japanese, given its pitch accent properties, e.g., Sato et al., 2007), again with the same training protocols, and see if the influence of individual differences changes.

Yet another possible explanation for the difference between the present study and that of Sadakata and McQueen (2013) lies in the nature of the stimuli. It may simply be the case that there are more individual differences in the way pitch is processed than in the way timing is processed. A number of studies suggest that pitch and timing information follow different processing pathways. For example, the ability to process rhythm information can be impaired while that to process pitch information remains intact (Alcock et al., 2000a,b) and vice versa (Hyde and Peretz, 2004). Furthermore, there may be genetic differences that determine individual differences in processing pitch information (Dediu and Ladd, 2007; Dediu, 2013) but perhaps not timing information. These factors may interfere with the process of category formation in different ways. A systematic investigation to establish which processes are shared across different speech features would be beneficial.

Individual differences are ubiquitous in speech learning (Strange and Dittmann, 1984; Bradlow et al., 1997; Hanulikova et al., 2012). A number of predictors that account for such individual differences have been identified and tested, such as aptitude (Wong and Perrachione, 2007; Perrachione et al., 2011; Ingvalson et al., 2014), working memory (Linck et al., 2013), age (Giannakopoulou et al., 2013), and neural correlates of pitch perception (Wong et al., 2007a, 2008; Chandrasekaran et al., 2012; Sheppard et al., 2012). Based on these features, learners are often classified into so-called good and poor learners. A critical and important question here is whether there is a way to support a learner who is predicted to be one of the poor ones. The current study suggests that one solution would be to adjust the level of variability in training materials to each individual’s level. Another way would be to identify the main predictor of individual differences in learning and then to directly train that feature. For example, recent studies (Cooper and Wang, 2013; Ingvalson et al., 2013) demonstrated that training listeners to perceive pitch directions prior to lexical tone training considerably helps low-aptitude perceivers to improve learning of non-native tonal categories.

In conclusion, the present results, taken together with those of Sadakata and McQueen (2013), suggest that it is not necessarily the case that high-variability training is the most effective way for listeners to learn non-native speech categories. For easier-to-learn categories, high-variability training may well be the most effective approach. But when categories become harder to learn, as when Dutch listeners have to adjust how they process pitch information in order to learn Mandarin lexical tone categories, high-variability training appears to help listeners who are good at speech perception, but makes things harder for those who are less good at speech perception.

## Conflict of Interest Statement

The authors declare that the research was conducted in the absence of any commercial or financial relationships that could be construed as a potential conflict of interest.
